# Retraction: Clematichinenoside Serves as a Neuroprotective Agent Against Ischemic Stroke: The Synergistic Action of ERK1/2 and cPKC Pathways

**DOI:** 10.3389/fncel.2021.699518

**Published:** 2021-06-04

**Authors:** Frontiers Editorial Office

The journal retracts the 12 January 2016 article cited above.

Following publication, it has been brought to our attention that several figures included in the article above appear identical to figures published by the authors in a separate article, “The neuroprotective effect of a novel agent N2 on rat cerebral ischemia associated with the activation of PI3K/Akt signaling pathway” (doi: 10.1016/j.neuropharm.2015.02.022), published in Neuropharmacology, February 2015. Specifically, the Bax-sham and Bax-AR(32 mg/kg) panels in Figure 2A of the Frontiers in Cellular Neuroscience publication appear identical to the D and F panels in Figure 5C of the Neuropharmacology publication, respectively. The TUNEL-AR(16 mg/kg) panel in Figure 2A of the Frontiers in Cellular Neuroscience publication also appears identical to panel C in Figure 4A of the Neuropharmacology publication.

The authors concur with the retraction and sincerely regret any inconvenience this may have caused to the reviewers, editors, and readers of Frontiers in Cellular Neuroscience.

